# From reflexivity to anti-reflexivity: climate change polarization in the United States

**DOI:** 10.3389/fsoc.2026.1743306

**Published:** 2026-04-01

**Authors:** Emily Swanson

**Affiliations:** Department of Sociology and Criminology & Law, University of Florida, Gainesville, FL, United States

**Keywords:** anti-reflexivity, climate change, partisanship and environment, political polarization, reflexivity

## Abstract

Climate change is an urgent issue that experts across disciplines and borders have been attempting to bring to the forefront of public discussion for decades. Understanding the socio-political and ideological factors influencing public perspectives on climate change has become critical in modernity, where reflexivity—the vigilance and response to environmental risk—and its counterforce, anti-reflexivity, shape how individuals and institutions react to climate change. There has been little synthesis of the empirical literature on reflexivity and anti-reflexivity regarding American public perceptions of anthropogenic climate change. It is crucial to initiate dialogue among these empirical pursuits. This paper responds to this call by analyzing the empirical literature on reflexivity and anti-reflexivity in relation to perspectives on climate change. This analysis concludes with a conceptual framework and pathways for future research, contributing to the discussion of public attitudes toward climate change in the United States.

## 1 Introduction

Human activities, such as the burning of fossil fuels, generates greenhouse gases, including carbon dioxide, methane, chlorofluorocarbons, and nitrous oxides, which become overly concentrated in the Earth’s atmosphere (Soeder, 2025). These rampant emissions, combined with decreased albedo from melting ice caps and reduced carbon sequestration from deforestation and warming seas, drive climate change (Cochard, 2013). Despite the scientific consensus on climate change, public and political responses remain far from unified. Over time, the nation’s bifurcation and gradual shifts in perspectives have become increasingly distinct (Oreskes, 2004). In one stream of empirical observation, political elites’ responses and messaging tend to normalize climate denial. Conversely, Sun et al. (2025) demonstrate that an increase in “elite cues” of climate change denialism can paradoxically trigger a backlash. Understanding the socio-political and ideological factors influencing public perspectives on climate change has become critical in modernity, where reflexivity—the vigilance and response to environmental risk—and its counterforce, anti-reflexivity, shape how individuals and institutions react to climate change.

The United Nations Framework Convention on Climate Change (UNFCCC) and the Paris Agreement have set ambitious climate goals: to hold the global average temperature increase to well below 2 °C above pre-industrial levels and to pursue efforts to limit it to 1.5 °C (UNFCCC, 2015). To meet these goals, collective, consistent, and just action is essential. Yet despite its outsized contribution to global emissions (13.49% in 2021), the United States continues to experience significant political resistance to climate action (Scott, 2023). Building the political will to address the multifaceted ecological and social ramifications of climate change requires understanding these underlying ideological dynamics (Gillard et al., 2016).

The majority, 58%, of Americans believe that climate change is happening and is human-caused, with 29% believing climate change is due to natural cycles rather than human activities, and only around 13% do not think climate change is happening at all (Leiserowitz et al., 2025). A significant body of literature explores American climate denialism and skepticism, distinguishing between trend skepticism (denial of climate change itself), attributive skepticism (denial of human causation), and impact skepticism (doubt about negative consequences) (Schmid-Petri et al., 2015). Researchers have also examined the factors that motivate belief in anthropogenic climate change, support for environmental movements, pro-environmental behaviors, and climate policy. Two central theoretical and empirical frameworks—reflexivity and anti-reflexivity—help explain why some believe in anthropogenic climate change and support climate action, while others resist it. This literature review outlines the history of this polarization, examines theoretical frameworks, synthesizes key empirical findings, illustrates a conceptual framework, and proposes directions for future research. This paper addresses two calls from established researchers in the area, one from Boström et al.’s (2024) article which draws attention to the gap of dialogue between studies of reflexivity and anti-reflexivity toward the crisis of climate change, and the second from McCright’s A. M. (2016, p. 187) systematic literature review, which calls for “continu[ing] our trajectory from descriptive to more explanatory work, designing more theoretically driven studies.” Further, this paper is not a systematic literature review, but rather a conceptual and theoretical starting point to identify lines of dialogue that can emerge when investigating reflexivity and anti-reflexivity simultaneously, as a quasi-dialectic, in understanding what the field has developed in these spheres of knowledge.

## 2 Historical overview

### 2.1 Climate science, activists, and politics

Investigating the United States’ environmental political trajectory reveals how reflexive and anti-reflexive tendencies have developed over time through the interaction of science, activism, and partisan ideology. The scientific community and environmental movements of the 1970s brought the urgent existential threat of climate change to the forefront of public awareness. Initially, the United States faced little apparent political divide regarding climate change and environmental protection policies. There was ostensibly bipartisan agreement that human activities were driving unprecedented global warming, necessitating action (Dunlap and Allen, 1976). In 1974, *Congressional Quarterly* staff writers noted, “The environmentalists in Congress cut across the lines of most traditional alliances—party affiliation, conservatives and liberals, regional interests” (Dunlap and Gale, 1974, p. 671). In his 1970 State of the Union address, Republican President Richard Nixon said, “The great question of the Seventies is… shall we make our peace with nature and begin to make reparations for the damage we have done to our air, to our land, and to our water” (Green, 1996, p. 418). He then signed the Clean Air Act of 1970 and established the Environmental Protection Agency (EPA), among numerous other pro-environmental policies (Green, 1996).

However, following the presidency of Jimmy Carter, a countermovement, ignited by the Republican Ronald Reagan administration and continued by conservative political elites and think tanks, soon challenged this political consensus, questioning the previously accepted scientific position on anthropogenic climate change (Jacques et al., 2008). During the rollbacks of the Reagan administration, a rupture occurred in the brief period of bipartisan environmentalism, as neoliberal policies exemplify an anti-reflexive position, prioritizing laissez-faire market-oriented policies over environmental accountability. This represented a significant shift within much, though not all, of the Republican Party.

As environmentalist movements highlighted scientific findings that demonstrated urgent issues arising from the Right’s deregulation and the repeal of environmental protections, along with their making federal lands available for private use and reforms to the EPA’s enforcement capabilities, this conservative countermovement responded with various forms of literature and media to create an alternative narrative to the science (Jacques et al., 2008). Public support for pro-environmental social movements remained strong in the 1980s, causing the countermovement to struggle (McCright and Dunlap, 2010). Ultimately, the countermovement moved away from directly opposing regulations and institutions like the EPA, or from targeting specific environmentalists in politics, to undermining environmental/impact science itself (McCright and Dunlap, 2010). By the 1990s, a significant political divide emerged among U.S. representatives over climate change.

### 2.2 Political divide in recent decades

Recent decades have revealed a deepening of the political trench, with the rhetoric of anti-reflexivity becoming increasingly concentrated alongside a party line in conservative discourse and policies. In 2011, only 48% of the American public believed there was a scientific consensus on climate change, revealing an opportunity for science communication to better inform yet also presented an opportunity for disinformation (McCright and Dunlap, 2011a). Notably, in 2015, Republican Senator James Inhofe of Oklahoma (and chair of the Senate Environment Committee) brought a snowball onto the Senate floor, claiming global warming as “The greatest hoax ever perpetrated on the American people” (Woolf, 2015). Bernie Sanders, a Democratic senator from Vermont, conversely, has spoken out about the 97% scientific consensus on anthropogenic climate change (Nuccitelli, 2014).

This juxtaposition epitomizes the party divide on climate change in contemporary American politics. According to data collected by the Yale Program on Climate Communication in 2024, most of the registered voters in their sample (*n* = 890) thought the development of clean energy should be a “high” or “very high” priority for the President and Congress. Still, there remains a stark divide by political party identity, with liberal Democrats reporting “high” or “very high” at 93%, moderate/conservative Democrats at 87%, liberal/moderate Republicans at 46%, and only 26% of conservative Republicans. There has also been a downward trend for the Republican group as a whole since 2018 (Leiserowitz et al., 2025). The United States has lacked consistency in its commitment to global initiatives, most famously, the Paris Agreement, with its stance shifting depending on the political party in office. These foundational uncertainties not only altered how environmental hazards and the severity of climate change are socially constructed and understood but also laid the groundwork for today’s pronounced polarization and contention surrounding climate science and environmental policy.

## 3 Literature review

### 3.1 Anthony Giddens’ concept of reflexivity

To further understand these dynamics of climate science, environmental activism, and politics in shaping the American public’s views and concerns about climate change, the literature review will first highlight an influential theoretical framework for analyzing how social practices, institutions, and individual members are continuously formed as they respond to new information. In examining Giddens’ theory, we identify the mechanisms that generate reflexivity and lay the groundwork for later in the paper to conceptualize its counterforce, anti-reflexivity, in contemporary American environmental politics. As Boström et al. (2024, p. 476) note, “A topic that deserves further attention is the relation between reflexivity and anti-reflexivity. There is promising research on anti-reflexive forces and trends, which could be fruitfully applied to the topic of knowledge resistance, climate denial, and similar fields.” Due to the limited studies conducted specifically in the United States, this paper relies on empirical tests of these theories, especially reflexivity, from other countries in the global North (e.g., the United Kingdom, Canada, Australia, and Germany).

Although these countries differ culturally, socially, and politically from the U. S., they may have similar motivators and obstacles to reflexivity, as well as forces of anti-reflexivity that are part of international climate change denial interests, such as the fossil fuel industry. This aligns with one of the few other existing articles that examines both reflexivity and anti-reflexivity by McCright et al. (2016a), which finds “across countries, the strength and consistency of ideology-based predictors vary by the mobilization and strength of the climate change denial countermovement.” While there are more studies on anti-reflexivity among the United States general population, few specifically examine reflexivity. There is also notable work from Germany, the United Kingdom, and Australia on anti-reflexivity that contributes to the literature. The transferability of these studies is a limitation in reflecting exactly what is occurring in the idiosyncratic context of contemporary U.S. politics. Nonetheless, it is a useful place to begin conceptualizing how to frame studies in the U.S. that examine both sides of response, reflexivity, and anti-reflexivity. This could be particularly the case, as social media platforms have become a major source for how Americans get their news and knowledge of the world (Pew Research Center, 2025). English is either the dominant spoken language or, in the case of Germany, the second most spoken language in the studies used from outside the U.S., and these ideas are transmitted, even siloed into echo chambers online, where reflexivity and anti-reflexivity come to exist transnationally (European Commission, 2024). While the use of social media to communicate and further polarize these ideas helps illustrate the application to the US context, it is notable that this paper draws on studies conducted outside the US, and as future research examines this area, a systematic literature review will be helpful in generating a more fully encompassing theory and framework.

The concepts of reflexivity, institutional reflexivity, and self-reflexivity originate from sociologist Anthony Giddens’s theory on the central distinction between modernity and traditional society in his book *Modernity and Self-Identity: Self and Society in the Late Modern Age,* published in 1991. The original definition of reflexivity by Giddens, often cited in the Environmental and Resource Sociology literature, is: “Social practices are constantly examined and reformed in the light of incoming information about those very practices, thus constitutively altering their character” (Giddens, 1991, p. 38). He defines institutional reflexivity as “the regularised use of knowledge about circumstances of social life as a constitutive element in its organisation and transformation” (Giddens, 1991, p. 20). Giddens (1991) even applies the concept to the very formation of self and the construction of identity as a demarcation of modernity from traditional society, writing, “The self is seen as a reflexive project, for which the individual is responsible. We are, not what we are, but what we make of ourselves (p. 75).” Identity itself becomes a reflexive process in modernity, extending this conceptualization of reflexivity to the multi-scalar. With the rise of science and technology, as well as changes in how we live, there have been greater and fundamentally altered risks since the Industrial Revolution (Beck, 1992). In modernity, we must manage these risks and navigate through the world, forming the self and constructing an identity around these decisions.

Reflexivity is an essential construct in the paradigm of ecological modernization, which developed in the 1980s, as it entails recognizing and adapting to the risks of climate change, particularly at the institutional and organizational levels, to decouple economic growth and development from ecological degradation. Ecological modernization scholars, such as Mol and Spaargaren (2000), argue that reflexivity can lead to solutions within the system itself, operating at an institutional level, analyzing governance such as the role of civil society and market mechanisms. According to Mol and Spaargaren (2000, p. 88), “Solutions to the detrimental consequences of modernization (among which environmental risks have a prominent place) are to be found in a radicalization of, rather than a breaking away from, modern institutions and practices.” The existence of both production science (e.g., new technologies such as electric cars) and impact science (e.g., studies on the health and environmental effects of the latest technologies produced) would suggest this is occurring at least in these arenas (Dunlap, 2014).

The reflexivity mechanism in modernity may be stifled by those with material and ideological interests in maintaining current transportation, industry, energy, and dominant agri-food systems. To further understand reflexivity, it is crucial to examine empirical studies that investigate how reflexive processes are enacted within various social practices. Empirical work provides valuable insights into how reflexivity influences policy views, personal and organizational behavior, and public attitudes towards climate change. By analyzing empirical evidence, we can evaluate the effectiveness of reflexive approaches in promoting belief in anthropogenic climate change and in supporting climate policy and examine their connections to anti-reflexivity.

The review approach for collecting studies that have engaged with the empirical conceptualizations of reflexivity and anti-reflexivity included a search of the Google Scholar database. The search parameters include the time frame 1990–2024, the English language, accessibility at the author’s institution, and an assessment of the connection to the topic, identifying studies that varied (e.g., scale, methodology, targeted population) from the reflexivity and anti-reflexivity literatures to include for analysis. This was so that the studies covered would provide depth to support the development of a conceptual framework that is not just any one kind of study from either literature, but rather representative of the ways in which these concepts have been investigated. The search terms included “reflexivity + climate change” and “anti-reflexivity + climate change.” Empirical studies on anti-reflexivity that covered a greater variety in forms of data, quantitative (surveys) and qualitative (content analysis), whereas reflexivity was qualitative, but varied in kind, such as including specifically looking at real-world interactions and conditions, speaking to stakeholders, observing the process, and including case studies.

## 4 Empirical studies of reflexivity

### 4.1 Strands of reflexivity

The literature on reflexivity as it pertains to human relationships with the environment, including the interest of this paper, anthropogenic climate change, takes various forms depending on the level of operation and, when combined, are central to developing a conceptualization of reflexivity at multiple levels of analysis. The strands of reflexivity this paper draws on in the current literature include the original conception imagined by Giddens’ work referenced above (Giddens,1991), which is further developed in Beck et al.’s (1994) reflexive modernization, and is incorporated into Mol and Spaargaren (2000) ecological modernization theory. More recent and individual applications of reflexivity have been developed by Archer (2003) and Hamilton (2022), respectively. Studies on reflexivity range from the individual (e.g., Davidson, 2012) to the mesoscopic organizational level (Haverkamp, 2017). For Giddens (1991), the self and institutions can engage in reflexivity under the conditions of modernity; they are, as his broader theory of structuration suggests, co-constitutive, the process in one is what gives form to the other.

Margaret Archer’s reflexivity has been applied to various fields of research, focusing on the formation of beliefs and behaviors of individuals ranging from studies on healthcare (Rybczynska-Bunt et al., 2024) to socioeconomic inequality (Baker, 2019). Central to these theories is the undercurrent developed by Giddens (1991) that integrates structure and agency to address what incites the reflexive process, in this case, in the interest of confronting the realities of anthropogenic climate change. Notably, Archer’s (1995) concept of reflexivity also presents a shift from Giddens, as it critiques Giddens for its nebulous character, whereas the development of her theory positions more of a dualism than integration, lifting up the concreteness of agency, which is important in how the literature bridge can develop here in considering how some people, organizations, and institutions develop reflexivity to climate change and seek to curtail its effects, whereas other instantiations are anti-reflexive. Hamilton’s (2022) development of emotional reflexivity helps inform this, specifically in relation to climate change, by explaining how individuals become engaged through emotions, which, in the process of engaging emotions (acknowledgement and expression), motivate action and agency. Reflexivity literature encompasses various levels of analysis and assumptions regarding the structure-agency debate, both of which contribute to and complicate our understanding of reflexivity, which is an important aspect for future research to consider.

According to Giddens (1991), reflexivity “involves the ability to recognize the role of the industrial capitalist system in creating crises of modernity such as climate change and to address such issues via institutional transformations and individual change.” Their work, which furthers the original reflexivity theory of Giddens is developed into “reflexive modernization” wherein it suggests there was a first modernity (“simple modernity”), characterized by industrialization, the construction of nation-states, and class-oriented structures, along with the grand narrative of technological and scientific progress. The rational process leads to modernity reflexively examining itself (“reflexive modernization” or using the terminology of Beck, “world risk society”) and its own processes as they lead to environmental destruction and enhanced environmental risks (e.g., pollution, rising seas) (Boström et al., 2016). Giddens (1991) combine the theories of risk society, institutional reflexivity, and cultural reflexivity to argue that modernity has developed the ability to critique itself within its own framework. This leads to a social world of reflexive modernity, where modernization that turns back on itself becomes the central focus of societal reform, not just abstract progress and unrestrained economic growth.

The principal methodology for studying the occurrence of reflexivity has been semi-structured in-depth interviews along with participant observation. One such study examining climate change-related reflexivity, conducted by Davidson in 2012, is based on interviews with two communities in Alberta, Canada. Importantly, all the interviewees were professionals or public servants in environmental decision-making within their respective communities. Davidson (2012) categorizes five participants as “meta-reflexives” who have a strong moral responsibility to mitigate climate change. However, while none of the interviewees denied climate change, there was significant variation in the levels and types of concern they expressed. The “meta-reflexives” were very concerned about the systemic effects of climate change, and all were highly concerned about it posing serious threats, as evidenced by examples from the interviews, specifically those related to food security and violence. The non-meta reflexives did not deny climate change, but they did not take any particular actions, unlike the meta-reflexives. Interestingly, the meta-reflexives were more apprehensive about the future. It was further found in Davidson’s (2012) analysis of the interviews that social networks influence the frequency of conversations about climate change, the tone of those conversations, and the behaviors that arise.

Davidson and Stedman (2017) found that a predisposition to reflexivity increases openness and the effort toward climate change mitigation policies and behaviors. Both studies utilize Margaret Archer’s theory of four modes of reflexivity: fractured reflexivity, communicative reflexivity, autonomous reflexivity, and meta-reflexivity, with an emphasis on the latter, as this is the mode of reflexivity they argue is most critical in responding to climate change. Meta-reflexivity had a larger influence on whether participants engaged in pro-environmental behaviors, even compared to other well-established predictors in the literature (education, political ideology, and perceived threat level of climate change).

Reflexivity has been further conceptualized by Hamilton (2022), who studies “emotional reflexivity” in the United Kingdom. The data was collected at workshops (Carbon Literacy Project and Work that Reconnects) and through interviews with the participants and facilitators. Hamilton (2022) argues that ignoring, not processing, or not acknowledging the emotions conjured by climate change “contributes to emotional paralysis and systems of socially organized denial.” For Hamilton, emotional reflexivity refers to how people acknowledge, reflect on, and engage with their feelings about climate change.

In this 2022 study, Hamilton looks to understand emotional reflexivity, how people cultivate it, and how the public can further mobilize it to take collective action against climate change. Specifically, they find that emotional reflexivity comes from awareness and acknowledgment of emotions, which facilitates engagement in climate change, and “expression and movement of emotions,” which resulted in the ability of participants to change their relationship with their emotions, leading to what Hamilton characterizes as a more sustained awareness of climate change. Further, in Hamilton’s (2022) study, they find that although reflexivity can generate a “deep determination,” the lack of these practices led to “defensive coping, ambivalence and vacillation, which impeded active engagement over time.” Despite these relatively positive results of this reflexivity, power dynamics may alter effectiveness at the community and organizational levels. This finding is worth considering in the context of the subsequent two U.S.-based studies, which examine community project developments and how power dynamics affect reflexivity, or even act as a force for anti-reflexivity.

Haverkamp’s (2017) study highlights the power dynamics that complicate or even inhibit reflexivity as the concept shifts from analysis of the self to the organization. Their study site was an urban and coastal region of Virginia that was and continues to experience climate change-related flooding. Through interviews and participant observation of community adaptation planning, they found that exclusionary practices exist and are motivated by power and security concerns. Consequently, project management lies with technocratic elites, which is unconducive to community involvement and environmental justice (Maguire and Lind, 2004). These findings suggest that procedural justice is a prerequisite for reflexivity at the community climate-resilience planning level. Through the interviews and observation of the forums, Haverkamp finds that the technocratic experts they interview and observe in this forum could benefit from critical reflexivity to recognize the structures and power dynamics present in the development of the adaptation project planning.

In the observation of the adaptation forums, Haverkamp (2017) finds that those in attendance do not reflect the diversity of the stakeholders, finding them to be from various institutions, including FEMA, NOAA, College of William and Mary—“80 well-educated, employed, and predominantly White participants… With few exceptions, community outsiders, the general public, and elected officials were not in attendance, nor were they invited. Forum demographics are characteristic of both a highly technical, as well as an elite gathering of individuals—all of whom were in attendance to collectively respond to climate impacts in the Hampton Roads region” (Haverkamp, 2017, p. 2682). Haverkamp’s study is particularly revealing because it further conceptualizes reflexivity, from the self to how the organizational response to climate change (adaptation strategies), to how experts [adding to Cunliffe’s (2004) “reflexive practitioner”] can be reflexive to power. “Reflexivity should be made critical, questioning issues of power and privilege such as: what knowledges underpin ideas of risk and vulnerability, power differentials in stakeholder engagement, and historically rooted social inequalities” (p. 2689). This critical approach to reflexivity is still underexplored in the literature. It offers scholars a valuable opportunity to consider their own reflexivity in their research and more broadly, within other organizations as they develop and execute adaptation strategies for climate change. This approach could also help mitigate political polarization related to anthropogenic climate change and the adaptation or mitigation strategies used to manage its impacts in communities. These power dynamics observed by Haverkamp (2017) that lead to exclusion may constrain the spread of reflexivity.

In their research on a wind turbine energy infrastructure development site in Northwestern Michigan, Howell (2012) using discourse analysis, finds that while the ideas of Beck’s risk society are plentiful in the documents and media coverage, views of reflexivity are not quite there yet, as reflexive modernization or ecological modernization theorists might claim or hope. There are multiple rationalities presented, but none of them are science or denialism, which Howell categorizes as “alternative science,” except for in the starting narrative, “when concerned citizens are focused most on the potential health impacts of large wind turbines, are competing scientific camps established, and this is only in reference to the issue of turbine set-backs.” It is perplexing that the risk society is manifesting in these discourses, and yet reflexivity is not occurring. Howell (2012) posits that this is due to conflicting knowledge claims, with the conservative parties’ lack of openness to transforming their ontological views, or changing policies and practices when critiqued, leading Howell to call for a separation between the concepts of risk society and reflexive modernization as they pertain to studies in energy and infrastructure projects. There is a disconnect between what theories of reflexivity suggest should happen at the individual level and in these project developments, and what actually occurs. There are possibilities for reflexivity, particularly at the personal level, but it is not inevitable.

While empirical studies of reflexivity reveal conducive conditions for reflexivity to occur at multiple scales, such as moral responsibility, emotional engagement, and institutional openness, they also demonstrate barriers to reflexivity. Despite its potential, reflexivity is not present in all contexts and often fails to materialize when confronted with the inertia of systemic power structures. Sociologists have theorized this due to a counterforce to reflexivity (Gleeson, 2000; McCright and Dunlap, 2010; Givens et al., 2020). The anti-reflexivity thesis elucidates American conservatives’ climate change denialism, stemming from organizational-level resistance that aims to create a sense of uncertainty about the science among the general public (Dunlap, 2013; Dunlap and McCright, 2012). The following sections will examine the rise of the anti-reflexivity thesis and consider the empirical studies by scholars who posit its effects as explanatory for the political divide in American politics toward anthropogenic climate change.

### 4.2 The rise of the anti-reflexivity thesis

Conservative political elites and other interest groups, most saliently the fossil fuel industry, benefit from the “manufacturing of doubt” concerning the scientific consensus of climate change (Lewandowsky et al., 2012). McCright A. (2016) conceptualizes anti-reflexivity as “the climate change denial countermovement as a collective force defending the industrial capitalist system against claims that the system causes serious problems.” Where impact science and the social movements of environmentalism call for reform or revolution (usually the latter), the forces of anti-reflexivity in the political, economic, and eventually cultural realms of society pushed by conservative entities allow anti-reflexivity to persist instead of confronting risks and considering one’s behavior or society’s behaviors, the alternative narrative of the unknown or skepticism can lead to a reflexive ignorance that qualms doxastic anxiety. Lewandowsky et al. (2013) found that participants underestimated the scientific consensus on climate change, with most believing there was a 70% consensus among scientists, whereas the reality was a 97% consensus.

The anti-reflexivity thesis is “a perspective that challenges the widely accepted view known as Reflexive (or Ecological) Modernization, which assumes that employment of scientific information—along with technological innovation and the market—allows modern societies to solve environmental problems without major modifications of their economic systems and growth trajectories (Dunlap, 2014, p. 1).” The environmental Kuznets curve suggested that, while economic growth initially increases environmental degradation, there is a turning point at which, through technological and social reflexivity, the destruction decreases (Lynch and Long, 2024). The anti-reflexivity thesis can partially explain this critique, which is often leveled at ecological modernizationists. If the social and political will that upholds the market system is not geared toward this decoupling, then widespread evidence of decoupling will not occur until reflexivity takes hold.

Early studies to test anti-reflexivity occurred at the political level, for example, in Congress. These early studies initially yielded contradictory findings. Some suggested that political officials showed a split in voting on environmental issues by political party, with Democrats much more likely to support environmental reform policies than Republicans (Dunlap and Allen, 1976; Kamieniecki, 1995). However, other studies found little to no statistically significant influence of party affiliation on the American general public’s views on environmental policies. Research by Pierce and Lovrich (1980) demonstrated that environmental issues were not well integrated into the mass belief system for most of the public. However, among the “attentive” and “activist” niche groups, differences in views on environmental regulation were evident, similar to those among legislators and party officials, with political party affiliation influencing perspectives. Ostensibly, this marks the earliest instances of Congress, legislators, and their most ardent supporters towing the party line on environmental issues. From there, the countermovement forms and disseminates this framing of skepticism to the broader general public.

One of the first studies to explore anti-reflexivity was published in 2000 by Gleeson, who posits that anti-environmentalist social movements fostered skepticism of science that explicitly demonstrates the ecological destruction caused by industrial capitalism (i.e., impact science). That same year, McCright and Dunlap (2000) analyzed publications from 14 conservative think tanks produced between 1990 and 1997. These publications relied on peer-reviewed papers from a small group of contrarian scientists, along with non-peer-reviewed works that claimed there was weak evidence for global warming, asserted that the “net effect of global warming” would be beneficial, and argued that policies aimed at reducing global warming would cause more harm. These early studies on the activities of conservative think tanks and the Republican Party, including these publications and media outlets, reveal an organizational-level countermovement spurred as a response to the environmentalist movements.

One of the early tests aimed at determining whether the “cleavages” among political elites were also reflected in the United States general public was conducted by Dunlap et al. (2001), who found that this was the case. The disinformation around climate science from the Republican Party’s political elites proved effective in reframing the threat posed by climate change. The 2000 Gallup “Earth Day” survey data analyzed in the study revealed overall support for the Environmental Movement, but identification as a Republican and conservative ideology significantly reduced support, as did other measures such as association with the environmental movement, self-identification as an “environmentalist,” etc. Interestingly, political party identification was as influential as the strongest indicator of political ideology on the outcome variables. Although statistically significant, the coefficients for political variables were relatively weak. They write:

“…The relatively limited efficacy of the four political variables plus key demographic variables in explaining variation in support environmentalism is disappointing…in the contemporary United States, environmental issues seem even less politically consensual than they were in the early 1970s.” (p. 44)

The later study, published in 2008 by Jacques, Dunlap, and Freeman, found that among their sample of 141 English-language books on environmental skepticism published between 1972 and 2005, 92% were associated with conservative think tanks. These books were published in high volume in 1992, the same year as the Rio de Janeiro Earth Summit. When considering the findings of Jacques et al. (2008), McCright and Dunlap (2000), and Dunlap et al. (2001) together, the division that began to emerge in the general discourse becomes sensible, as there was significant pushback functioning as a countermovement to environmentalists, especially in response to the Earth Day summit, which garnered considerable public attention and support.

With the increasing anti-reflexivity among conservative political elites and stakeholders in preserving neoliberal ideology becoming evident in the wider public, it is crucial to consider how climate change is framed in everyday conversations and media (Stecula and Merkley, 2019). Researchers have sought to understand how framing the message of climate change produces anti-reflexivity in individuals. The next section of the literature review on anti-reflexivity will examine the empirical findings from framing analysis, followed by the quantitative studies that have tested anti-reflexivity.

### 4.3 Anti-reflexivity framing studies

The framing of information plays a crucial role in how individuals receive and evaluate knowledge claims. Social science researchers studying climate change have considered how framing influences respondents’ perspectives. Using an experimental design where subjects faced three conditions framing climate change as a risk to the environment, health, and national security, Myers et al. (2012) examined how different audience segments responded. Among the six audience segments representative of the United States public, they found that framing climate change as a public health risk elicited the strongest emotional responses and supported promoting climate change mitigation policies and strategies, particularly those related to climate change adaptation. This is consistent with Howell’s (2012) study on the framing of reflexivity, as it reveals that reflexivity is not occurring; rather, the elements of Ulrich Beck’s risk society are present. Moreover, this finding aligns with Hamilton’s (2022) later development of emotional reflexivity, as the audience processes the emotional risks associated with their health.

To test the influence of anti-reflexivity, McCright et al. (2016b) employ an experimental design featuring four “promising” frames of anthropogenic climate change: economic opportunity, national security, Christian stewardship, and public health, alongside counter-frames that promote anthropogenic climate change denialism. They find that the four positive frames of anthropogenic climate change have little influence on the subjects’ beliefs about the issue, but the denial counter-frame significantly reduces their belief. The denial counter-frame diminishes the subjects’ “belief about the veracity of climate science, awareness of the consequences of ACC, and support for aggressively attempting to reduce our nation’s GHG emissions in the near future.” Further, the effect of the denial counter-frame was more substantial among subjects who identified as conservative than among those who identified as moderate or liberal.

Küppers (2024) investigates the Alternative for Germany (AfD) case to explore the relationship between ideology and climate change skepticism by analyzing text from the party’s membership magazine. Consistent with findings out of U. S. studies, such as Givens et al. (2020), Küppers finds that the AfD frames climate change skepticism as aligned with its free-market ideology. The underlying message is that climate change policies conflict with Germany’s national interests. Furthermore, the AfD contextualizes climate change skepticism within their broader host ideologies, specifically radical right-wing ideologies. Although Küppers finds that the AfD often emphasizes the “core people” in its general framing, populism does not prominently appear in its portrayal of climate change and countermovement as a force of anti-reflexivity.

### 4.4 Quantitative studies of anti-reflexivity: what are the most influential independent variables?

There is an evident correlation between political party affiliation and a tendency to reject climate change entirely or to suppress the role of human activities in driving it (Uscinski and Olivella, 2017; Layzer, 2014; Dietz, 2020; Jacques et al., 2008; McCright and Dunlap, 2011a). This has also been consistent with research by Tranter (2011) in Australia, which found that political ideology was the most influential factor in whether respondents believed in climate change. Additional variables outside of political affiliation that have been shown to predict belief in climate change include region in the United States, rural/urban locale, sex, age, income, religion, education, political ideology, and race (Hornsey et al., 2016).

In a longitudinal study examining public trust in science, which analyzed data from 1974 to 2010, Gauchat (2012) found that Republicans in the early 1970s demonstrated the highest level of trust in science. Liberals and moderates maintained stable levels of trust in science throughout the study period. Respondents identifying as Republicans were the only ones to experience a decline in trust in science over the study period. By the 2000s, Republicans exhibited the lowest level of trust in science recorded in the study. In their 2013 study, McCright et al. demonstrate more nuance and complexity in the conservative distrust of science. Their analysis finds that while conservatives distrust impact science, they trust production science at higher rates than liberals. The primary explanation for this political polarization and Republican climate change denialism comes from the anti-reflexivity thesis posed by McCright and Dunlap (2011b), which is “a perspective that attributes conservatives’ (and Republicans’) denial of anthropogenic climate change (ACC) and other environmental problems and attacks on climate/environmental science to their staunch commitment to protecting the current system of economic production (Dunlap, 2014).” The reliance on the current economic structure, such as the dependence on fossil fuels, leads to a united political push to obfuscate the scientific findings on the effects of anthropogenic climate change.

In their McCright and Dunlap (2011b) study, which utilized data from the Gallup Survey conducted between 2001 and 2010, McCright and Dunlap found that White conservative men are significantly more likely than other groups to deny climate change. This difference becomes even more pronounced as they self-report higher levels of understanding regarding global warming. Although this study is somewhat dated, it illustrates how deeply embedded this skepticism has become within a specific demographic of the United States public compared to earlier studies and connects to other parts of identity, like religiosity and gender. Further examining specific sociodemographic characteristics, such as McCright and Dunlap’s analysis of gender and race/ethnicity, Givens et al. (2020) analyze data from the Intermountain West region of the United States. They find strong support for the anti-reflexivity thesis, with the respondents’ views on the free-market economy (neoliberalism) being the most influential among the various outcome variables related to environmental concerns. Moreover, supporting the free-market economy, identifying as a Republican, and holding a more conservative political ideology are all negatively correlated with worries over global warming and climate change. Research by Givens et al. (2020) raises questions about whether a decline in neoliberal ideology, such as during an economic downturn, where faith in that system could be decreased, would lead to a corresponding rise in the belief that human activities drive climate change or if the belief stays consistent with party ideology generating a sort of cognitive dissonance that the subsequent study discussed by McCrea et al. (2015) examines.

McCrea et al. (2015) investigate an integral question that requires clarification in the context of the United States and further research to better understand climate change skepticism and its relationship to political views. Using longitudinal survey data from Australia, they sought to address how the relationship operates, whether skepticism or denialism of climate change motivates voting behavior, or whether voting behavior influences their views of anthropogenic climate change. In focusing on the post-election attitudes, they find that voting influences climate change skepticism more than climate change skepticism influenced the original voting behavior. “The findings also suggest that changes in the levels of skepticism are partly due to the way that political parties distinguish themselves in their position and subsequent policies on climate change. Thus, in countries where there is more bipartisan political support for responses to climate change, we might expect to find lower and more stable levels of climate change skepticism in electorates” (McCrea et al., 2015, p. 1329). In dialogue with other studies, this could suggest that the political polarization of climate change stems from the anti-reflexivity of the Right’s political party and how voting connects the individual more closely with that group identity, leading them to adopt the ideas of that group. McCrea et al. (2015) also suggest the counter is true, that “For example, if a person votes for a green political party though still holds some skepticism about climate change, he or she may then experience cognitive dissonance and subsequently change his or her attitude to be less skeptical about climate change after voting green” (p. 1309). This is quite divergent from the self-reflexivity theorized by Giddens.

**Figure 1:**
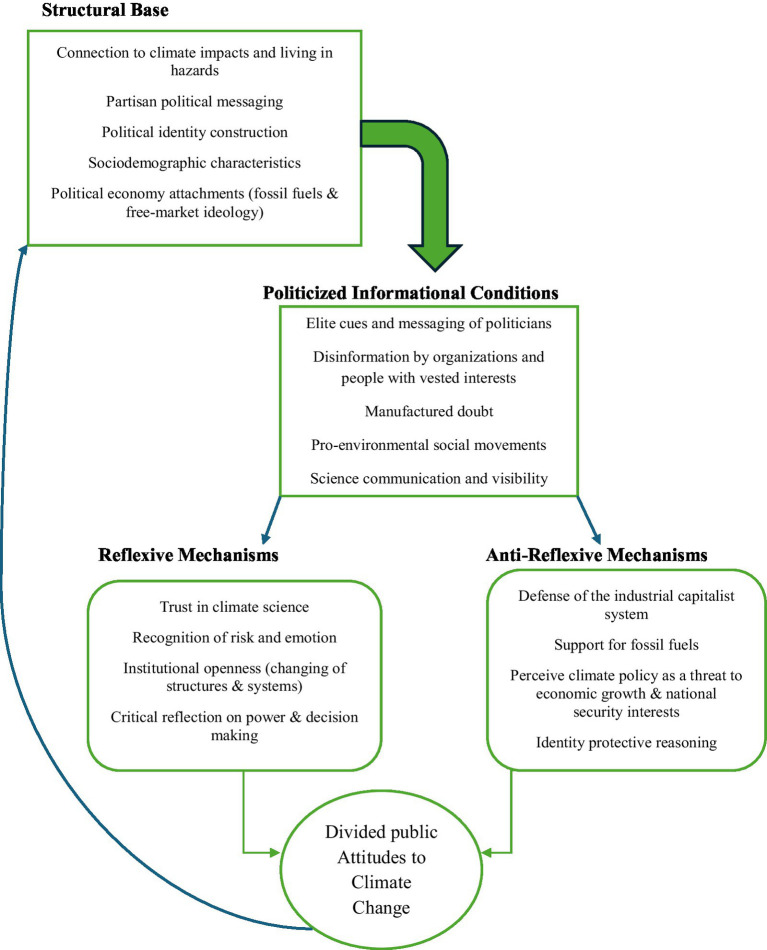
The duality in perceptions of reality: countervailing forces model.

The empirical research on reflexivity and anti-reflexivity has yielded an expansive view of the influences and formations shaping the American public’s perceptions of climate change, but noteworthy gaps and areas of ambiguity persist. Hitherto, studies have illuminated the sizable impact of political ideology, economic interests, and framing on views of climate change, particularly the skepticism that has permeated the Republican Party. Still, critical questions regarding the interactions between reflexivity and anti-reflexivity in individuals and communities remain unanswered. Figure 1 visualizes the countervailing forces as a conceptual framework, illustrating a potential mechanism for viewing the connections and feedback from structural bases to the politicization and polarization of information, and the reflexive and anti-reflexive pathways to public attitudes.

## 5 Directions for future research

To broaden the literature’s knowledge and dialogues, future research should address these limitations, such as investigating the simultaneity of these forces in the public or even within individuals, and their interactions with larger structures, and how these forces are enacted during systemic crises or failures. The following conclusion outlines potential pathways for advancing these lines of inquiry. The sociological climate change literature would benefit from studies examining the relationship between reflexivity and anti-reflexivity (Boström et al., 2024). Research tends to focus on either reflexivity or anti-reflexivity, so these conversations have largely been kept separate. As illustrated in this review of the literature, there are also fundamentally differing approaches and methodologies, with reflexivity being studied using qualitative interviewing, focus groups, case studies, and field observations, and anti-reflexivity, while in the early days, it used media and content analysis, tends more towards quantitative data analysis using survey data. Moreover, empirical studies of reflexivity (Davidson, 2012) cite the work of McCright and Dunlap’s anti-reflexivity more than the anti-reflexivity scholars cite studies of reflexivity. As they are counterforces, it would be beneficial to use the same dataset to examine the variables that lead to either reflexivity or anti-reflexivity, thereby gaining a better comprehension of political polarization and how Americans think about climate change. In fostering belief and acceptance of climate change, these reflexive practices at various levels can help achieve international climate goals.

Beyond the importance of cultivating a collective consciousness of reflexivity in modernity, studying reflexivity and anti-reflexivity together can enable continued theoretical development and discussion between ecological modernization and treadmill theories. Anti-reflexivity can help explain, at least in part, the opposition inherent in these two paradigms. It finds partial truths in both views. While ecological modernization theory may argue that a green transition is possible through reflexivity, the treadmill of production theory suggests that these reflexivity tools are intentionally suppressed to maintain growth and profit motives.

The extant literature on reflexivity and anti-reflexivity presents numerous interesting findings, with several areas remaining underexplored, and questions remain unanswered. Anti-reflexivity posits that material, ideological investment, and attachment to existing fossil-fuel-dependent capitalist systems, without commitments to reducing climate change, prevent the reflexivity conceptualized by reflexivity theorists and by scholars of ecological modernization. The findings of Givens et al. (2020) suggest that free-market ideology has the strongest influence on environmental views. It would be useful to examine whether system- or structural-based failures (such as market crashes like the 2008 Great Recession) undermine trust in the conservative countermovement that promotes climate change skepticism and denial, since this could theoretically impact individuals’ material interests, if not their sense of identity.

Here, I propose three research pathways for future empirical studies to consider exploring:

- *Pathway one* could empirically evaluate the motivations of reflexivity and anti-reflexivity by examining sociodemographic and ideological variables to determine which increases each type of phenomenon—for example, testing working-class people who identify as Republican and those who identify as Democrats with those who would have a material stake in the current political economic system, potentially using occupational category, income, etc. this could reveal the anti-reflexivity influence as particular or stronger for those who personally benefit economically and with status while controlling for political party affiliation. Then, using measures like those available in the General Social Survey, evaluate their perceptions and trust in institutions (such as the scientific community, business leaders, or politicians) across cross-sections in 2006 and 2010 to understand the forces of reflexivity and anti-reflexivity, and whether education can influence this association. Research that investigates and tests both phenomena can unify these isolated discussions and potentially create a more dynamic and nuanced framework for studying American denial and skepticism toward anthropogenic climate change. Further, in studying adaptation and infrastructure plans for climate change or creating climate-resiliency, participant observation researchers should look for agents of reflexivity and of anti-reflexivity in these community or organizational meetings and development operations. Potential guiding research questions could be:

◦ *What is the role of material, ideological, and identity investment in the current system in hindering individual-level reflexivity?*
◦ *What is the role of education in producing reflexivity or intervening in anti-reflexivity forces?*

- *Pathway two* investigates how economic crisis promotes either reflexivity (because of distrust in a failing system and recognizing the crisis inherent in an un-reflexive political-economic order) or anti-reflexivity (framing from the political party one identifies with) to see if this leads to increasing polarization, with a possible guiding question:

◦ *Does an economic recession, such as in 2008, increase polarization of views on anthropogenic climate change?*

- *Pathway three* replicates the study by McCrea et al. (2015) in Australia to see whether, in the US, the pattern of voting behaviors influencing views on anthropogenic climate change holds, or if the influence works the other way. This could help inform the understanding of how anti-reflexivity is inculcated into an individual through their identity formation with the political party they vote for.
